# Filling gaps – a case study in building advocacy capacity in the health promotion workforce

**DOI:** 10.1177/17579759241246778

**Published:** 2024-05-06

**Authors:** Melissa Stoneham, Lee Coller, Jacqueline Napolitano, Megan M. Scolyer, Christina Pollard

**Affiliations:** 1 Public Health Advocacy Institute Western Australia, Curtin University, Perth, Australia; 2 Goulburn Valley Public Health Unit, Shepparton, Australia; 3 Gateway Health, Wodonga, Australia

**Keywords:** advocacy (including media advocacy), health promotion, workforce development

## Abstract

Public health advocacy is a fundamental part of public health and health promotion practice. However, gaps exist in the provision of public health advocacy knowledge and skill acquisition both in the tertiary environment and within ongoing professional development programmes. The Goulburn Valley Public Health Unit partnered with the Public Health Advocacy Institute to build the skills of 49 public health and promotion professionals in their regions, to enable them to lead an advocacy project that aimed to promote state-wide initiatives. This involved a series of face-to-face skills-based public health advocacy workshops and post workshop e-mentoring. Results included the creation of locally relevant public health advocacy projects and a community of practice.

## Why is advocacy important to public health and health promotion?

Public health advocacy is a fundamental part of public health and health promotion practice (1). The World Health Organization (WHO) describes advocacy for health as a combination of individual and social actions designed to gain political commitment, policy support, social acceptance and systems support for a particular health goal or programme (2). It is included as one of three pillars within the WHO’s Ottawa Charter for Health Promotion, being to advocate, enable and mediate (2). In 2016, the International Union for Health Promotion and Education (IUHPE) published a set of Core Competencies and Professional Standards, which underpin the registration and accreditation of health promotion professionals. The nine competencies specify the knowledge, skills and performance criteria required to demonstrate acquisition of the core competencies. One of the nine core competencies is the ability to advocate for health, requiring an applicant to competently advocate with, and on behalf of individuals, communities and organisations to improve health and wellbeing and build capacity for health promotion action (3). This competency is reflected in the broader public health sector contemporary expectation that a public health practitioner can advocate for healthy public policies and services that promote and protect the health and wellbeing of individuals and communities (4).

Advocacy is an important tool to build skills of the health promotion and public health workforce in order to strengthen health programmes, services and policies leading to improved health and societal outcomes (5). Yet, an audit of advocacy curricula in undergraduate and postgraduate Australian university courses identified that one-third of all identified degrees do not include advocacy as part of core curricula, with advocacy rarely included in degree learning outcomes (6). Other studies have identified that once graduates enter the workforce there are limited professional development oppor-tunities in advocacy, and a limited number of advocacy organisations exist to provide expert guidance (7,8).

Both health promotion and public health are ambitious health-related enterprises of the 20th century, with advocacy seen as a key strategy (9). However, there is a critical need to build the capacity of our current and future public health workforce in public health advocacy as it has been demonstrated that advocacy can achieve systemic change by addressing the social determinants of health (1,10).

These issues highlight the need for external courses to be offered to health promotion and public health professionals to address current curriculum gaps and ensure that the workforce is equipped to understand what advocacy is, how it can add value to existing programs and how best to evaluate its effectiveness.

To address this gap, the Public Health Advocacy Institute (PHAI) based at Curtin University developed and facilitated a series of skills-based advocacy workshops with the aim to increase skills and address the necessity of public health advocacy among health professions. These interactive, action-focused workshops added a new dimension to how public health and health promotion professionals are trained in advocacy, and aimed to strengthen the workforce and build healthier communities. The case study that follows outlines the process and impact within six months of the advocacy training being completed. This case study could be replicated in other regions or countries.

## Impetus to change – the Goulburn Valley Public Health Unit

The Goulburn Valley Public Health Unit (GVPHU) is one of nine public health units across metropolitan and regional Victoria, representing seven local government areas across north-eastern Victoria. The unit governed by Goulburn Valley Health (GV Health) was originally established in late 2020 in response to the COVID-19 pandemic and is funded by the Victorian Department of Health. The vision of the GVPHU is to facilitate equitable, accessible health services and consistency of care through empowering people in the community to optimise their individual wellbeing and drive better health outcomes across the region. The GVPHU focuses on five key areas of work, being Health Protection, Intelligence Systems and Digital Innovation, Public Health Integrated Planning and Programs, Enga-gement, Communications and Capacity Develop-ment and Emergency Management (11). In 2023, in partnership with a community Health Service in North East Victoria, Gateway Health, the GVPHU embarked on an advocacy project that aimed to address the need for advocacy skills to assist in the systems changes needed to improve the community’s health. There was also a systems perspective understanding of the complexities of the causes and consequences of health promotion issues among some of the staff owing to their involvement in group model building to address obesity (12). Within the project objectives were two key foci, including the building of capacity, skills and knowledge of advocacy within the health promotion and prevention sector, and the integration of advocacy into everyday work. The GVPHU and Gateway Health partnered with PHAI to achieve these two objectives.

## Conceptual framework for the advocacy training

Advocacy is the active support of a cause and aims to develop consensus driven key messages, programmes and policies to promote the cause. Public health advocacy efforts can take on many forms, employing a range of strategies that aim to influence and advance evidence-based policymaking to improve health and wellbeing for individuals and populations. Whatever the form, public health advocacy requires a clear call to action and needs to be solutions based. A critical point of difference between advocacy and more generic public health strategies is the need to maximise support by strategically planning the ways the cause will be argued, including giving special attention to counteracting or reframing any strengths of potential opponents’ arguments (1,13). Effectiveness of public health advocacy can be demonstrated in changes to tobacco control legislation in Australia, where advocates used strategies such as collaboration and promoting new evidence to achieve continued government investment in no smoking campaigns and forming a chorus of health groups strongly urging the Government to place graphic images of smoking health outcomes on every pack of cigarettes sold in Australia. Advocacy strategies such as these were successful in securing public and political support for tobacco control legislation, policy and programme support.

Over the past few decades, several authors have described the processes of policy development and implementation in terms that are helpful for understanding public health policy and advocacy (14,15). Public health advocacy products, processes and participants are part of a multidimensional effort that has frequently defied diagrams and clear conceptualisation (16). To address this, the PHAI developed a conceptual framework for the process of public health advocacy that is consistent with advocacy theory and is based on a cyclical process. Within the model, there are eight stages and within each stage, there are multiple steps or components. Each stage is logically sequential, reflecting the fact that attention shifts conceptually – temporally and, over the short term, from one stage to another throughout the public health advocacy journey. The eight stages answer the following questions, and examples of how the questions are addressed and how this model is used, were discussed in the advocacy workshops.

1. What is the issue or ‘advocacy ask’?: The advocacy ask needs to be stated clearly , describing what is hoped to be solved through policy change. During the workshops, participants were asked to identify local issues where policy change could increase health outcomes. These issues were noted, discussed and in some cases combined (e.g. obesity and junk food). A group consensus activity identified issues that workshop participants would address in small groups. Issues were then magnified, and each group identified a specific policy change that formed their advocacy ask.
2. What evidence is needed to advance advocacy and how robust is it? Time did not permit workshop participants to extensively seek all available and emerging evidence relating to their advocacy ask. Instead, discussions revolved around what evidence is, ensuring its credibility and how to present it to enable influence.
3. Who or what is the opposition? The workshop provided case studies on how advocacy issues will have allies and opponents. Identifying potential opposers to the advocacy ask, pre-empting the evidence the opposition may use, anticipating the type and degree of pushback and allocating resources to combat opposition were discussed. The workshop participants used this theory to identify potential opposition related to their advocacy ask.
4. Who are you seeking to influence? Advocacy is a form of influence, and clearly articulating who the policy ask is targeting in order to change policy is a critical step. At this stage, the workshop examined internal, external, political and media and community advocacy as key drivers for influence and participants identified who their advocacy ask was seeking to influence.
5. Who will be your coalition or partners? The workshop focused on the idiom of ‘being inside the tent’ and the power that can be generated from coalitions. Formal and informal coalitions, the benefits of partnerships and power were discussed, before participants identified potential partners for their advocacy ask.
6. What is your key message? At the end of the advocacy journey, what is the one question you want answered? In advocacy, there is an expectation that to be successful the coalition must ‘sing from the same hymn sheet’ to ensure a memorable and repeatable message and advocacy ask. Participants were given the opportunity to develop key messages relevant to their advocacy ask.
7. Which advocacy strategies are best suited to achieve your advocacy ask? An extensive array of advocacy strategies was showcased in the workshops and aligned with those in the Advocacy in Action Toolkit (1). Participants were given time to identify innovative advocacy strategies, which formed the basis of participant presentations at the completion of the workshop.
8. How will you measure how or if your advocacy programme has achieved the advocacy ask or policy change? The importance of, and challenges associated with, measuring change from advocacy were identified. A guide to evaluating advocacy efforts as outlined in the Advocacy in Action Toolkit (1) was provided and participants were asked to consider their evaluation tactics.

**Figure 1.**
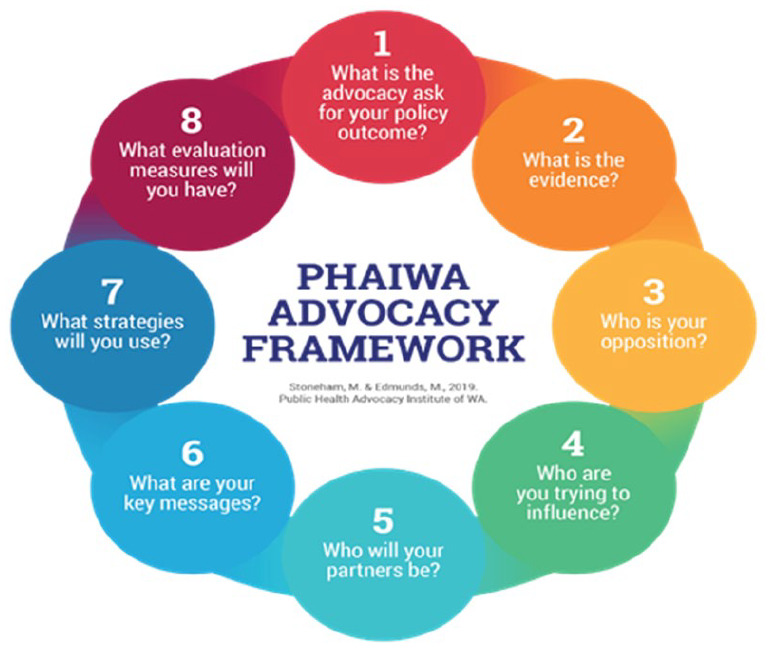
Public Health Advocacy Institute advocacy model. PHAIWA: Public Health Advocacy Institute of Western Australia

This model is shown in Figure 1.

The workshops utilised a constructivist or enquiry-based approach to learning, which enabled the participants to apply the theory taught to local community priorities, rather than on previously run state or national advocacy examples. The topics generated by the participants reflected local social and political issues within participant-led small group activities, and encouraged practical problem-solving rather than abstract or deductive thinking.

## Background on the advocacy workshops

Across the Hume region, the health promotion workforce is engaged in RESPOND (17), a community-based systems dynamics approach to understanding the complexities of and addressing childhood obesity. In February 2023, Gateway Health received a request from the Wodonga RESPOND (brains-trust) community to provide advocacy training. Gateway Health and GVPHU formed an official partnership and agreement to develop and implement the advocacy project. This involved regular meetings and a pre-advocacy survey to identify baseline knowledge and skills in advocacy. The survey targeted health prevention staff and Community Health–Health Promotion workforce in the Hume region to gain an understanding of the level of need and scope for the advocacy training.

To support the GVPHU in meeting their objectives, it was agreed to conduct three skills-based public health advocacy capacity-building courses in 2023; two in Benalla, Victoria and one in Wodonga, Victoria. Two of these workshops targeted health professionals, with the third targeting community members. The first health professional workshop was one day in duration and the second workshop ran over two days. The final one-day workshop catered to community members who were interested in pursuing advocacy goals. It was further agreed that PHAI would offer e-mentoring sessions for up to five campaign advocacy groups following the workshops. The e-mentoring was an opt-in activity, to enable workshop participants to actively use the knowledge, skills and partnerships developed at the workshops.

The advocacy model illustrated in Figure 1 was used as a framework for the course development. The model provided a mechanism for working through typical steps in advocacy strategy development, allowing for tailoring to meet local needs. The workshop facilitated a process where participants gained consensus on which local issues would be the focus of their advocacy plan for the workshop duration. In general, each workshop addressed four local issues. Sufficient time was allocated to ensure a solid theoretical understanding of each stage within the advocacy model, and was supported with the provision of examples of good practice followed by group work to develop local strategies for each stage. The workshops culminated with a presentation from each group acting out the advocacy plan to the whole group, with presentation styles embracing uniqueness and flair.

## Participants and satisfaction levels

Over the three workshops, a total of 49 participants attended. The aim of these workshops was to facilitate discussion and build advocacy skills among different preventive actors in the Hume region in Victoria, with a view to develop a shared understanding of the current challenges and opportunities in this area. The three workshops were planned to integrate active learning, group participation, fun and relevant case studies while considering the diversity of contexts, needs and approaches. A workshop evaluation was completed by 34 participants (69%), with 56% attending the two-day workshop and 45% attending the one-day workshop. The effectiveness data were similar across the one- and two-day workshops and indicated that the workshop aims were met. Of the respondents, all (100%) stated the workshop was excellent or very good and 16 (47%) participants advised that they would be interested in post-training mentoring. Every respondent (100%) indicated that having completed the workshop, they held more awareness about the development of an advocacy project or campaign, and 32 respondents (94%) advised that after having completed the workshop, they had the skills to develop an advocacy project or campaign as part of their role.

## Encouraging sustainability – post workshop mentoring

Five advocacy campaigns emerged from the workshops. They identified local priorities and included:

- Addressing alcohol and gambling harm in Wodonga through policy (local government level);
- Mandating of Food and Drink Policy in school canteens – Victoria;
- Stakeholder engagement for both healthy eating and physical activity pillars of Community Health–Health Promotion plan;
- Increasing the visibility of the health promotion workforce through key messaging;
- Lobbying to gain political support for the Albury Wodonga Regional Food Share and Route 66 parklands trail.

The effectiveness of e-mentoring in the field of public health advocacy has been established, with studies identifying the value of such a programme to increase public health advocacy knowledge, skills, confidence and experience, and build public health networks (8,9). Studies also identified through participant feedback an increased confidence to integrate public health advocacy into their work practice and an awareness of the role of advocacy in the achievement of public health objectives (9). Given these findings and the ability for professional mentoring to stimulate the acquisition of theoretical knowledge, practical skills and encourage the application of the learnings (18) from the advocacy workshops, it was agreed that PHAI staff would e-mentor up to three people from each of the emerging advocacy projects developed during the workshops.

Over the six months post the advocacy workshops, a total of 11 professionals were involved in the e-mentoring programme, and nine unique e-mentoring sessions were conducted. Actions resulting from these sessions were diverse and included outcomes such as drafting alcohol policies, creating new networks and partners, encouraging submissions from community members and local professionals in response to a Community Impact Assessment for a packaged liquor outlet, creating consensus based advocacy campaign messages (#ThisIsPrevention; #HealthPromotionInMotion) and inspiring local community members to attend governance workshops with a view to running for local government elections.

The advocacy training also led into the development of a Hume advocacy Community of Practice (CoP), a collective of workshop participants who continue to meet following the advocacy workshops to progress advocacy ideas, provide support and build coalitions. The CoP has 24 members from 11 organisations and meets monthly. Overall, the advocacy training, the CoP and the mentoring programme increased engagement and partnerships with health promotion and prevention partners as well as the broader contribution to longer term capacity building in advocacy across the Region.

## Discussion

This paper describes the capacity building of local public health and health promotion staff to integrate public health advocacy within their current project planning and implementation.

As advocacy is not currently or routinely included within the core curriculum of many universities, some public health students are graduating without advocacy competencies. This has a knock-on effect, resulting in certain sectors of public health being insufficiently prepared to develop and deliver advocacy. This can stall public health and health promotion advances that seek to build consensus, seek policy change, or improve the health of populations. It can also affect professionals seeking accreditation under the IUHPE core competencies programme. It was observed that independent courses such as those that PHAI offers are critically needed to fill this gap and provide practical strategies to build the workforce capacity in advocacy. Building local capacity likely creates positive change within communities (12), and this project shows that providing capacity building in public health advocacy through skills-based workshops and e-mentoring can support the public health workforce to access and apply knowledge from PHAI’s experience in complex advocacy and community-based health promotion projects.

The absence of an advocacy model on which to base capacity building and planning of advocacy programs was identified. There has not been a widely used or accepted framework or model for describing and explaining how public health advocacy can add value to existing programmes. The PHAI framework, coupled with numerous relevant practice examples used to guide the advocacy training, filled this void and can support public health professionals to build advocacy capacity, ensuring their programmes, partnerships, policies and services are as effective as possible.

The advocacy workshops and post workshop mentoring provided a critical resource to build knowledge, skills and confidence for workshop participants to proactively engage in advocacy activities. The e-mentoring programme was effective in supporting participants to navigate advocacy in the real world. The mentoring relationships were clear regarding aims and expectations, had the support of senior managers and, because all participants knew the mentors and had established a rapport at the face-to-face workshops, there was a good ‘fit’ between the underlying ethos of health promotion, public health, advocacy and the model of mentoring. The diversity of actions resulting from the e-mentoring programme demonstrates the effectiveness of this approach.

The public health advocacy capacity building model represents one of the first sustained efforts to integrate advocacy capacity within the Hume region public health and health promotion workforce. The capacity building provided the opportunity for the GVPHU staff to work with local partners to support and plan informed and collaborative advocacy projects that aimed to enhance the health and wellbeing of their local communities. This was demonstrated by their ability to collectively identify and plan advocacy programmes focused on local priorities and the formation of the local community of practice. This programme of delivering tailored advocacy workshops in local communities, with follow-up e-mentoring to support emerging advocacy programmes which built upon the knowledge and skills developed at the workshops, serves as a model for ensuring advocacy is a core topic of professional development programmes. It also safeguards high quality, innovative and effective advocacy programmes and leadership that continue to advance the public health workforce.

## Conclusion

This paper has provided an example of establishing the capacity of public health advocacy within a regional Victorian public health workforce, and could be replicated in any region or country.

Despite advocacy being a core pillar and competency within the public health fields, public health advocacy is not well addressed in either university curricula or within ongoing professional development programmes, resulting in certain sectors of public health being insufficiently prepared to develop and deliver advocacy. This case study demonstrates that many public health professionals are interested to learn how to integrate advocacy into their mainstream duties and, when offered the opportunity, are enabled to forge local partnerships, coalitions and projects to progress locally relevant advocacy.

Providing staff development opportunities in public health advocacy can inspire partnerships within and across sectors to address complex public health issues.
